# Reports on Hospitals of the United Kingdom

**Published:** 1913-11-15

**Authors:** Henry Burdett


### REPORTS ON Hospitals of the United Kingdom.

By SIR HENRY BURDETT, K.C.B., K.C.V.O.

SERIES III.

### SOME COTTAGE HOSPITALS IN SUFFOLK AND CAMBS.

### WOODBRIDGE HOSPITAL AND SECKFORD DISPENSARY.

On our way from Ipswich to Lowestoft we passed through Woodbridge, in Suffolk (population 4,623), where we stayed to inspect the Woodbridge Hospital and Seckford Dispensary, established in 1861. It is a new building of red brick, with some architectural pretensions, and presents, exteriorly, a bright and modern appearance.

A Deserted Building.

We rang the bell and getting no answer we opened the door and went in. On the ground floor from the street is a dispensary, with good waiting-hall and consulting-room and a dispensary proper. Upstairs we found two wards with two beds in each and a nurses' room with the usual offices. The wards were unoccupied, some of the beds not even made up and none appeared to have been occupied recently. There were two cupboards, one of which contained some of the mackintosh cloaks of the nurses, and the other a very small stock of linen, chiefly towels. No one was to be found in any part of these buildings.

Returning to the ground floor and finding nobody we noticed a staircase leading to the basement. On going down the steps we found that this consisted of some rooms with beds, a good sitting-room looking out on to the garden, a larder, and other offices. No one was on this part of the premises either, in fact we had evidently come, for the first time in our experience, on a hospital which not only contained no patients but was entirely devoid of any living creature, human or animal.

No Sign of Life.

Written application has never produced a copy of the report, nor did the building contain one. Having examined every part of the building and everything it contained, there being nothing else to do, we departed. It would be interesting to hear an explanation from someone connected with this remarkable institution as to the nature of the work undertaken, the number of the staff, and the name of the officer in responsible charge. We were divided in opinion as to whether it was one of the old form of cottage hospitals, with a dispensary, which is worked by the parish nurse, whose duties lie chiefly outside the buildings, or whether the lowest part of the building is occupied by district nurses, or to what purpose this portion of the building is devoted. We have inspected many hundreds of institutions of all kinds during the last forty years, but never before had we found a deserted dispensary or sick house which had apparently no inhabitants and which was left, with every door unlocked, at the mercy of any casual stranger whose curiosity might lead him to turn the handle and walk through the building as we did at 11.30 a.m. on Tuesday, October 21, 1913.

### ST. LEONARD'S HOSPITAL, SUDBURY, SUFFOLK.

This hospital was founded in 1867, and enlarged in 1906 and 1912. It is a pleasantly situated hospital of fifteen beds, which affords evidence that a progressive spirit animates the committee. It was surprising and most encouraging to find, everywhere throughout the hospital proper, that someone with up-to-date knowledge and keenness for efficiency is responsible for its administration. We went over the whole building without discovering who that person was. Subsequently we had the pleasure of meeting the matron, the honorary secretary, and a member of the committee, when we found that the matron had been trained at Guy's, was keenly and self-denyingly attached to her work, of which she had a sound, practical knowledge. It appeared, too, that the committee were anxious to develop and maintain the institution as an up-to-date, highly efficient example of a smaller hospital of modern type.

Points and a Query.

The hospital occupies an elevated site, which is a good feature, but it necessarily contributes to make the building somewhat cold in the severer portions of each year. The large wards at one end are consequently rather cold sometimes, but as they contain good fireplaces there should be no difficulty in keeping them comfortable always.

The hospital has an excellent, well-lighted, well-proportioned, workmanlike theatre, but we doubt if it is entirely justifiable to keep the sterilisers in the neighbouring dispensary. One feature of this little hospital is an inner lobby where ample lockers have been fitted up so that each patient—all of whom pay something per week—may have an entire locker to put his or her clothes in. We presume that these clothes are disinfected and carefully examined in doubtful cases before being removed into the lockers.

A Chance for Two Families.

We observed amongst the generous supporters of this hospital the names of two well-known families—those of Quilter and Aird. If one or both of them were to reconstruct the east wing, giving it an ample verandah on the ground floor with access to the ward, and an upper storey with some additional small wards for acute medical or special operation cases when necessary, they would thereby get a better yield for their money and do more practical good than opportunity often offers to the benevolent.

We have long preached that the cottage hospital hygienically should be a centre of education for the inhabitants of the district it serves in matters of hygiene and health. Sudbury Cottage Hospital sadly lacks a well-kept, simple mortuary chamber, which, from its design and arrangements, would properly afford evidence of reverence for the dead. At present there is a lean-to shed which does not contain any supply of water or any drainage, and we are of the opinion, seeing the good quality of the work which the medical staff are doing in this hospital, that the mortuary should have attached to it a pathological chamber with every requisite for the best work.

### National Insurance as Incentive!

There can be no doubt that Sudbury is one of the smaller hospitals which should have its usefulness increased and its work extended through the National Insurance Act. The improvements we have suggested, if taken in hand, might be completed and in occupation before March 1917, when the effects of National Insurance will probably have reached the stage where they must be faced and provided for.

### Hospital Plans in Board Rooms.

We would suggest that a plan of the hospital, drawn to scale, should be placed in the room where the committee meet. It happened to be the day after "Pound Day" when we visited the hospital on October 20, 1913, and the success of this movement was testified by the fact that three thousand "pounds" in various forms were brought to the hospital. At a larger hospital 15,000 "pounds" are sometimes obtained.

We observe that the committee, like other committees, in their report intimate that there is a possibility that the hospital will derive some benefit under the Act with regard to insured persons who have no dependants. The Amending Act of 1913 has effectually checked the prospect of any benefit coming to voluntary hospitals through this channel.

### ROUS MEMORIAL HOSPITAL, NEWMARKET.

This hospital was founded in 1881, enlarged in 1897, and remodelled in 1906. The governing body consists of the committee of members of the Jockey Club, a sub-committee of whom constitute the management.

It contains fifteen beds and has about 220 patients a year, of whom one-third are out-patients. It is admirably equipped throughout, and is kept thoroughly up to date in every department. There are two wards, of which that for men contains eight beds and that for women four beds. The wards are attractive in appearance, light, airy, well furnished, beautifully kept; indeed, the whole hospital when we visited it was as spick and span as a new pin. It is true, we rejoice to say of this little hospital, that it contains everything that science can suggest for the most up-to-date and skilled treatment of its patients. The fact is that the authorities of the Jockey Club have only to be convinced that something is desirable to make their hospital better fulfil its purpose and they at once procure it.

### The Staff and Mr. Rothschild.

The staff are well provided for and have every comfort. The sanitary and other equipments are of the best, and therefore the simplest and plainest possible. The staff take a great interest in their work, and the *x*-ray department and other special departments are kept in constant work and in efficient working order.

We were pleased to hear that Mr. Leopold de Rothschild takes a great personal interest in this little hospital, as he does in many others, and that he is regarded with gratitude by the staff because he never fails to remember the hospital or to help in every emergency those in misfortune whom the hospital happens to touch in the course of its work.

There is one thing, however, which is worth careful consideration. The problem is how it would be possible to relieve the dullness and solitude which the nursing staff and the matron have to face very often. One remedy would be for the ladies who during considerable portions of the year make Newmarket their residence, with much pleasure and enjoyment, to put their heads together to arrange and see that as far as possible the nursing staff of the Rous Memorial Hospital are not forgotten, and that everything is done whilst they are in Newmarket to make residence at the hospital as little monotonous and solitary as possible. Of course, the staff is small, and this adds to the difficulty in one sense. Nurses as a class have much to do, and are particularly appreciative of a little kindness and thoughtfulness towards themselves, a fact which is to be commended to the consideration of all whom it may concern.

### An Additional Need.

The present matron is Miss A. Langridge, who was trained at the London Hospital, and had excellent experience in the duties of a matron, for she was one of the sisters working in the matron's office at the London, and there learned much that must prove of the utmost value to her in her work.

We venture to suggest for the consideration of the committee that they should devote a little attention to the mortuary building, with a view to providing, in connection with it, a chapel or view room, where the patients' friends could see their dead under the most sympathetic and reverent conditions. In a hospital as replete as the Rous Memorial Cottage Hospital is to-day, it seems a pity not to make its mortuary so complete as to secure that it should carry with it lessons which cannot fail to be of value to the community which the hospital serves.

**Some Essex Hospitals will be reported on next week.**

